# Local quantum thermal susceptibility

**DOI:** 10.1038/ncomms12782

**Published:** 2016-09-29

**Authors:** Antonella De Pasquale, Davide Rossini, Rosario Fazio, Vittorio Giovannetti

**Affiliations:** 1 NEST, Scuola Normale Superiore and Istituto Nanoscienze-CNR, Piazza dei Cavalieri 7, I-56126 Pisa, Italy; 2 ICTP, Strada Costiera 11, I-34151 Trieste, Italy; 3 Centre for Quantum Technologies, National University of Singapore, 3 Science Drive 2, Singapore 117543, Singapore

## Abstract

Thermodynamics relies on the possibility to describe systems composed of a large number of constituents in terms of few macroscopic variables. Its foundations are rooted into the paradigm of statistical mechanics, where thermal properties originate from averaging procedures which smoothen out local details. While undoubtedly successful, elegant and formally correct, this approach carries over an operational problem, namely determining the precision at which such variables are inferred, when technical/practical limitations restrict our capabilities to local probing. Here we introduce the local quantum thermal susceptibility, a quantifier for the best achievable accuracy for temperature estimation via local measurements. Our method relies on basic concepts of quantum estimation theory, providing an operative strategy to address the local thermal response of arbitrary quantum systems at equilibrium. At low temperatures, it highlights the local distinguishability of the ground state from the excited sub-manifolds, thus providing a method to locate quantum phase transitions.

## Abstract (web summary)

In thermodynamics, thermal properties of systems are obtained from averaging procedures which smooth out local details. Here, the authors introduce the concept of local quantum thermal susceptibility, a measure for the best achievable accuracy of estimation of temperature via local measurements.

## Graphical abstract

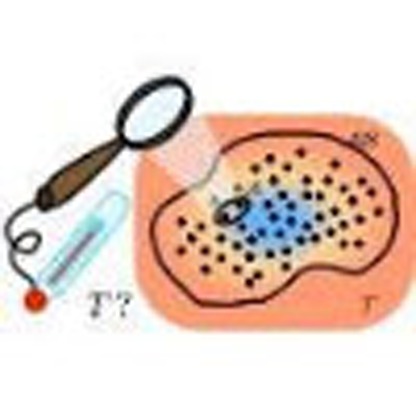

The measurement of temperature is a key aspect in science, technology and in our daily life. Many ingenious solutions have been designed to approach different situations and required accuracies^1^. What is the ultimate limit to the precision at which the temperature of a macroscopic state can be determined? An elegant answer to this question is offered by estimation theory^2^,^3^,^4^: The precision is related to the heat capacity of the system^5^,^6^.

In view of the groundbreaking potentialities offered by present-day nanotechnologies^7^,^8^,^9^,^10^,^11^,^12^ and the need to control the temperature at the nano-scale, it is highly relevant to question whether the heat capacity is still the relevant (fundamental) precision limit to small-scale thermometry. The extensivity of the heat capacity is a consequence of the growing volume-to-surface ratio with the size^13^. However, at a microscopic level such construction may present some limitations^14^,^15^. Moreover a series of theoretical efforts recently concentrated on a self-consistent generalization of the classical thermodynamics to small-scale physics, where quantum effects become predominant^16^,^17^,^18^,^19^,^20^,^21^,^22^. In particular, a lot of attention has been devoted to the search for novel methods of precision nanothermometry that could exploit the essence of quantum correlations^23^,^24^,^25^,^26^,^27^,^28^. In this context, the possibility to correctly define the thermodynamical limit, and therefore the existence of the temperature in the quantum regime, has been thoroughly investigated. It has been shown that the minimal subset of an interacting quantum system, which can be described as a canonical ensemble, with the same temperature of the global system, depends not only on the strength of the correlations within the system, but also on the temperature itself^29^,^30^,^31^. Using a quantum information-oriented point of view, this phenomenon has also been highlighted in Gaussian fermionic and bosonic states, by exploiting quantum fidelity as the figure of merit^32^,^33^. Furthermore, the significant role played by quantum correlations has been recently discussed with specific attention to spin- and fermonic-lattice systems with short-range interactions^34^.

In this paper, we propose a quantum-metrology approach to thermometry, through the analysis of the local sensitivity of generic quantum systems to their global temperature. Our approach does not assume any constraint neither on the structure of the local quantum state, nor on the presence of strong quantum fluctuations within the system itself. It is motivated by the observation that the temperature is a parameter that can be addressed only via indirect measurements, as it labels the state of the considered systems. Specifically, we introduce a new quantity that we dub local quantum thermal susceptibility (LQTS), according to the following scheme: Given a quantum system 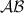 in a thermal equilibrium state, the LQTS 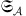 is a response functional, which quantifies the highest achievable accuracy for estimating the system temperature *T* through local measurements performed on a selected subsystem 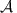 of 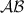 (see Fig. 1).

The LQTS is in general not extensive with respect to the size of 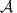, yet it is an increasing function of the latter, and it reduces to the system heat capacity in the limit where the probed part coincides with the whole system 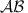. In the low-temperature limit, we shall also see that the LQTS is sensitive to the local distinguishability between the ground state and the first excited subspace of the composite system Hamiltonian. In this regime, even for a tiny size of the probed subsystem, our functional is able to predict the behaviour of the heat capacity and in particular to reveal the presence of critical regions. This naturally suggests the interpretation of 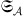 as a sort of mesoscopic version of the heat capacity, which replaces the latter in those regimes where extensivity breaks down.

## Results

### The functional

Let us consider a bipartite quantum system 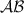 at thermal equilibrium, composed of two subsystems 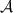 and 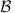, and described by the canonical Gibbs ensemble 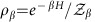. Here 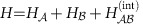 is the system Hamiltonian, which in the general case will include both local (that is, 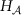 and 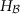) and interaction (that is, 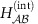) terms, while 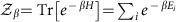 denotes the associated partition function (*β*=1/*k*_B_*T* is the inverse temperature of the system, *k*_B_ the Boltzmann constant, and {*E*_*i*_} the eigenvalues of *H*). In this scenario, we are interested in characterizing how the actual temperature *T* is perceived locally on 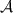.

For this purpose, we resort to quantum metrology^35^ and define the LQTS of subsystem 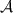 as


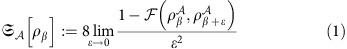


where 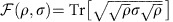 is the fidelity between two generic quantum states *ρ* and *σ* (refs 36, 37). The quantity (1) corresponds to the quantum Fisher information (QFI; refs 3, 4) for the estimation of *β*, computed on the reduced state 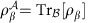. It detects how modifications on the global system temperature are affecting 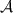, the larger being 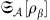 the more sensitive being the subsystem response. More precisely, through the quantum Cramér–Rao inequality, 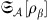 quantifies the ultimate precision limit to estimate the temperature *T*, by means of any possible local (quantum) measurement on subsystem 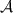. In the specific, it defines an asymptotically achievable lower bound,


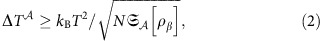


on the root-mean-square error 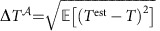 of a generic local estimation strategy, where *T*^est^ is the estimated value of *T*, 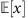 is the expectation value for a random variable *x* and *N* is the number of times the local measurement is repeated.

By construction, 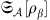 is a positive quantity that diminishes as the size of 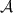 is reduced, the smaller being the portion of the system we have access to, the worse being the accuracy we can achieve. More precisely, given 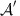 a proper subset of 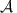, we have 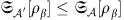. In particular, when 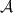 coincides with the whole system 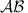, equation (1) reaches its maximum value and becomes equal to the variance of the energy,


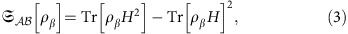


which depends only on the spectral properties of the system and which coincides with the system heat capacity^5^,^6^ (note that, rigorously speaking, the LQTS quantifies the sensitivity of the system to its inverse temperature β; the corresponding susceptibility to *T*=1/(*k*_B_*β*) gets a 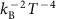 correction term, which also enters the standard definition of the heat capacity).

An explicit evaluation of the limit in equation (1) can be obtained via the Uhlmann's theorem^38^ (see the ‘Methods' section for details). A convenient way to express the final result can be obtained by introducing an ancillary system 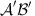 isomorphic to 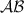 and the purification of *ρ*_*β*_ defined as


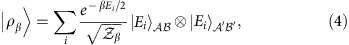


where 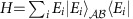 is the spectral decomposition of the system Hamiltonian. It can then be proved that


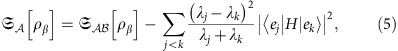


where {|*e*_*j*_〉} are the eigenvectors of the reduced density matrix 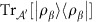 living on 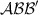, obtained by taking the partial trace of |*ρ*_*β*_〉 with respect to the ancillary system 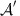, while {*λ*_*j*_} are the corresponding eigenvalues (which, by construction, coincide with the eigenvalues of 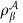).

Equation (5) makes it explicit the ordering between 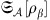 and 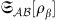: the latter is always greater than the former due to the negativity of the second contribution appearing on the right hand side. Furthermore, if *H* does not include interaction terms (that is, 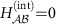), one can easily verify that 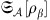 reduces to the variance of the local Hamiltonian of 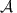, and is given by the heat capacity of the Gibbs state 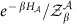 which, in this special case, represents 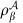, that is, 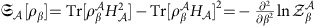. Finally we observe that in the high-temperature regime (*β*→0) the expression (5) simplifies yielding


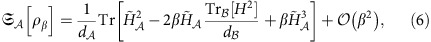


where 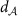 and 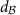 denote the Hilbert space dimensions of 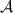 and 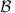 respectively, and we defined 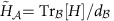 having set, without loss of generality, Tr[*H*]=0.

### A measure of state distinguishability

In the low-temperature regime, the LQTS can be used to characterize how much the ground state of the system 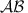 differs from the first excited subspaces when observing it locally on 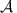. This is a direct consequence of the fact that the QFI (which we used to define our functional) accounts for the degree of statistical distinguishability between two quantum states (in our case the reduced density matrices 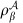 and 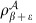) differing by an infinitesimal change in the investigated parameter (in our case the inverse temperature *β*). Therefore for *β*→∞, the LQTS can be thought as a quantifier of the local distinguishability among the lowest energy levels in which the system is frozen.

**Figure 2:**
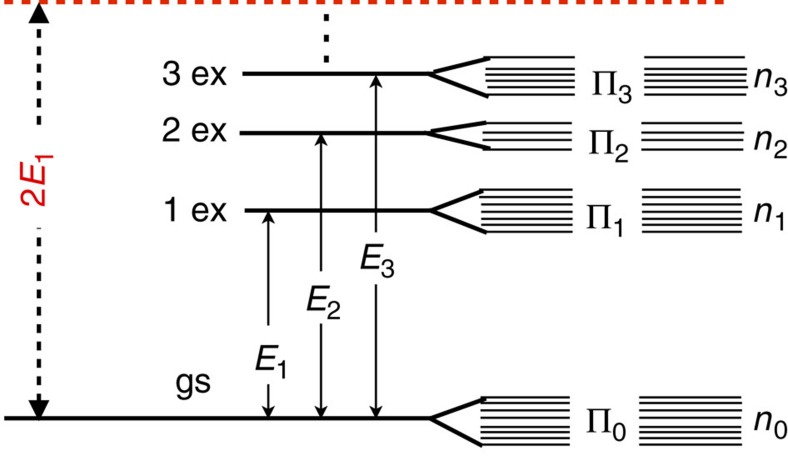
Energy spectrum of a quantum system. The figure provides a schematic representation of the low-energy spectrum for a generic many-body quantum system. For simplicity the ground-state (gs) energy *E*_0_ is set to zero. Here Π_*i*_ denotes the normalized projector on the eigenspaces of energy *E*_*i*_, which can be *n*_*i*_-fold degenerate.

To clarify this point, let us consider the general scenario depicted in Fig. 2, where we only discuss the physics of the ground state (with energy *E*_0_=0) and of the lowest excited levels with energy *E*_*i*_ bounded by twice the energy of the first excited level, *E*_*i*_≤2*E*_1_. The degeneracy of each considered energy eigenstate is denoted by *n*_*i*_. From equation (5), it then follows that up to first order in the parameter we get


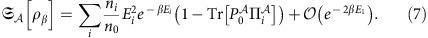


Here 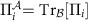, where Π_*i*_ is the normalized projector on the degenerate subspace of energy *E*_*i*_. Moreover, 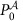 is the span of the local subspace associated to the ground state, that is, 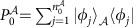 with 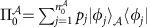 and 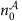 being the number of non-zero eigenvalues (*p*_*j*_>0) of 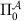.

Equation (7) can be interpreted as follows. Our capability of measuring *β* relies on the distinguishability between the states 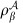 and 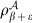, with *ɛ*<<*β*. In the zero-temperature limit, the system lies in the ground state and locally reads as 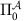, while at small temperatures, the lowest energy levels start to get populated. If their reduced projectors 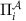 (*i*≥1) are not completely contained in the span of 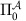, that is 
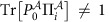
, there exist some local states whose population is null for *T*=0 and greater than zero at infinitesimal temperatures. Such difference implies that the first order in 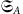 does not vanish. On the contrary, if the reduced projectors 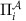 are completely contained in the span of 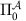, that is 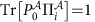, we can distinguish 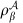 from 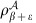 only thanks to infinitesimal corrections 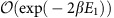 to the finite-valued populations of the lowest energy levels (see the ‘Methods' section for an explicit evaluation of the latter). In conclusion, the quantity 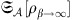 acts as a thermodynamical indicator of the degree of distinguishability between the ground-state eigenspace and the lowest energy levels in the system Hamiltonian.

### LQTS and phase estimation

A rather stimulating way to interpret equation (5) comes from the observation that, in the extended scenario where we have purified 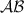 as in equation (4), the global variance (3) formally coincides with the QFI 
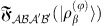
 associated with the estimation of a phase 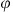 which, for given *β*, has been imprinted into the system 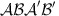 by a unitary transformation 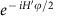, with *H*′ being the analogous of *H* on the ancillary system 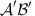, that is, 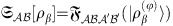 where 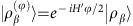 (refs 4, 35, 39). Interestingly enough, a similar connection can be also established with the second term appearing in the right hand side of equation (5): indeed the latter coincides with the QFI 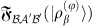 that defines the Cramér–Rao bound for the estimation of the phase 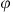 of 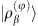, under the constraint of having access only on the subsystem 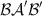 (that is, that part of the global system which is complementary to 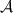). Accordingly, we can thus express the LQTS as the difference between these two QFI phase estimation terms, the global one versus the local one or, by a simple rearrangement of the various contributions, construct the following identity


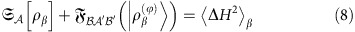


that establishes a complementarity relation between the temperature estimation on 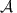 and the phase estimation on its complementary counterpart 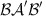, by forcing their corresponding accuracies to sum up to the energy variance 〈Δ*H*^2^〉_*β*_ of the global system (3).

### Local thermometry in many-body systems

We have tested the behaviour of our functional on two models of quantum spin chains, with a low-energy physics characterized by the emergence of quantum phase transitions (QPTs) belonging to various universality classes^40^.

Specifically, we consider the quantum spin-1/2 Ising and Heisenberg chains, in a transverse magnetic field *h* and with a *z* axis anisotropy Δ respectively,


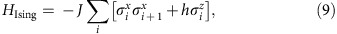



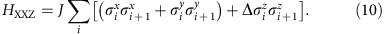


Here 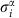 denotes the usual Pauli matrices (*α*=*x*, *y*, *z*) on the *i-*th site, and periodic boundary conditions have been assumed. We set *J*=1 as the system's energy scale. At zero temperature, the model (9) presents a 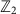-symmetry breaking phase transition at |*h*_c_|=1 belonging to the Ising universality class. The Hamiltonian (10) has a critical behaviour for −1≤Δ≤1, while it presents a ferromagnetic or antiferromagnetic ordering elsewhere. In the latter case, the system exhibits a first-order QPT in correspondence to the ferromagnetic point Δ_f_=−1, and a continuous QPT of the Kosterlitz–Thouless type at the antiferromagnetic point Δ_af_=1.

**Figure 3:**
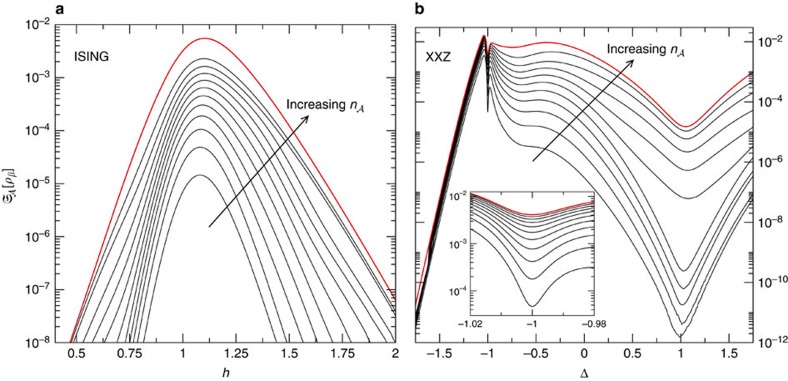
The LQTS in the Ising and the Heisenberg models at low temperature. We numerically computed the LQTS of equation (1) in the low-temperature limit for a chain with *L*=12 sites in the following two cases: (**a**) the Ising model as a function of the adimensional transverse field *h*; (**b**) the Heisenberg XXZ chain as a function of the anisotropy Δ. The uppermost (red) curve corresponds to the global quantum thermal susceptibility, that is the heat capacity. The other curves stand for different sizes 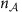 of the measured subsystem 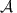 of 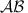 (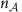 increases along the direction of the arrow). The inset in **b** magnifies the data around Δ=−1. In the XXZ model, the LQTS with 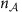
=1 can be proved to rigorously vanish. The inverse temperature has been fixed in both cases at *β*=9.

Figure 3 displays the small-temperature limit of 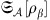 for the two models above, numerically computed by exploiting expression (21) in the ‘Methods' section. We first observe that, as expected, for all the values of *h* and Δ, the LQTS monotonically increases with increasing the number 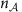 of contiguous spins belonging to the measured subsystem 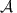. More interestingly, we find that even when 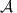 reduces to two or three sites, its thermal behaviour qualitatively reproduces the same features of the global system (represented in both models by the uppermost curve). In particular, even at finite temperatures and for systems composed of 12 sites, the LQTS is sensitive to the presence of critical regions where quantum fluctuations overwhelm thermal ones. The reminiscence of QPTs at finite temperatures has been already discussed via a quantum-metrology approach, through the analysis of the Bures metric tensor in the parameter space associated with the temperature and the external parameters^41^. The diagonal element of such tensor referring to infinitesimal variations in temperature, corresponds to the thermal susceptibility of the whole system. The latter quantity has been recently studied for the XY model^28^, showing its sensitivity to critical points of Ising universality class.

In the low-temperature regime, such global sensitivity can be understood within the Landau–Zener (LZ) formalism^42^. This consists of a two-level system, whose energy gap Δ*E* varies with respect to an external control parameter Γ, and presents a minimum Δ*E*_min_ in correspondence to some specific value Γ_c_. Conversely, the global heat capacity (3) may exhibit a maximum or a local minimum at Γ_c_, according to whether Δ*E*_min_ is greater or lower than the value of ΔE* at which the expression 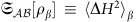 is maximum in Δ*E*, respectively. Indeed it can be shown that 〈Δ*H*^2^〉_*β*_ for a two-level system exhibits a non-monotonic behaviour as a function of Δ*E*, at fixed *β* (see the ‘Methods' section). Quite recently, an analogous mechanism has also been pointed out for the global heat capacity in the Lipkin–Meshkov–Glick model^27^. The LZ formalism represents a simplified picture of the mechanism underlying QPTs in many-body systems. However, by definition, the temperature triggers the level statistics and the equilibrium properties of physical systems. Therefore, both the heat capacity of the global system^5^,^6^ and the LQTS of its subsystems are expected to be extremely sensitive to the presence of critical regions in the Hamiltonian parameter space.

In the Supplementary Note 1, we performed a finite-size scaling analysis of 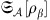 as a function of the size of the measured subsystem. For slightly interacting systems, one expects the LQTS to be well approximated by the heat capacity of 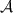 (at least when this subsystem is large enough). The latter quantity should scale linearly with its size 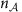. This is indeed the case for the Ising model (9), where a direct calculation of 〈Δ*H*^2^〉_*β*_ can be easily performed^28^. Our data for the scaling of the stationary points of 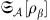 close to QPTs suggest that significant deviations from a linear growth with 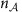 are present (see the Supplementary Fig. 1). This indicates that correlations cannot be neglected for the sizes and the systems considered here. A similar behaviour has been detected for the XXZ model, as shown in the Supplementary Fig. 2.

## Discussion

We have proposed a theoretical approach to temperature locality based on quantum estimation theory. Our method deals with the construction of the local quantum thermal susceptibility, which operationally highlights the degree at which the thermal equilibrium of the global system is perceived locally, avoiding any additional hypothesis on the local structure of the system. This functional corresponds to the highest achievable accuracy up to which it is possible to recover the system temperature at thermal equilibrium via local measurements. Let us remark that, even if in principle, the Cramér–Rao bound is achievable, from a practical perspective it represents a quite demanding scenario, as it requires the precise knowledge of the Hamiltonian, the possibility to identify and perform the optimal measurements on its subsystems, and eventually a large number of copies of the system. However, in this manuscript, we have adopted a more theoretical perspective, and focused on the geometrical structure of the quantum statistical model underlying local thermalization.

In the low-temperature regime, our functional admits an interpretation as a measure of the local state distinguishability between the spaces spanned by the Hamiltonian ground state and its first energy levels. Furthermore, we established a complementarity relation between the highest achievable accuracy in the local estimation of temperature and of a global phase, by showing that the corresponding accuracies associated with complementarity subsystems sum up to heat capacity of the global system. Finally, we considered two prototypical many-body systems featuring quantum phase transitions, and studied their thermal response at low temperatures. On one hand, we found that optimal measurements on local systems provide reliable predictions on the global heat capacity. On the other hand, our functional is sensitive to the presence of critical regions, even though the total system may reduce to a dozen of components and the measured subsystem to one or two sites.

Let us remark that most of the results presented herewith do not refer to any specific choice of the interaction Hamiltonian, 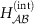 between 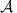 and 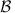. As an interesting implementation of our scheme, we foresee the case of non-thermalizing interactions^43^,^44^, whose potentialities for precision thermometry have been already unveiled.

We conclude by noticing that, while in this article we focused on temperature, the presented approach can be extended to other thermodynamic variables (like entropy, pressure, chemical potential and so on), or functionals^45^. In the latter case, quantum-estimation-based strategies, not explicitly referred to a specific quantum observable, but rather bearing the geometrical traits of the Hilbert space associated to the explored systems, may provide an effective route.

## Methods

### Derivation of useful analytical expressions for the LQTS

Let us recall the definition of the LQTS for a given subsystem 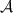 of a global system 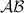 at thermal equilibrium:


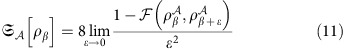


where 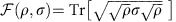 is the fidelity between two generic quantum states *ρ* and *σ*. According to the Uhlmann's theorem^38^, we can compute 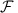 as


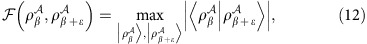


where the maximization involves all the possible purifications 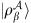 and 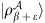 of 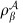 and 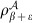, respectively through an ancillary system *a*. A convenient choice is to set *a*=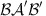, with 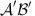 isomorphic to 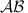. We then observe that, by construction, the vector |*ρ*_*β*_〉 of equation (4), besides being a purification of *ρ*_*β*_, is also a particular purification of 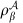. We can now express the most generic purification of the latter as


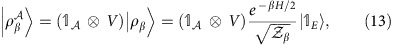


where *V* belongs to the set of unitary transformations on *a*, where 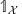 represents the identity operator on a given system 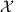, and where in the last equality we introduced the vector 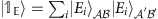, 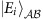 being the eigenvectors of *H*. We can thus write the fidelity (12) as


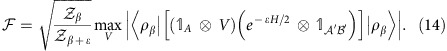


Since we are interested in the small-*ɛ* limit, without loss of generality we set *V*=exp(*i ɛ* Ω), with Ω being an Hermitian operator on the ancillary system *a*. It comes out that, up to corrections of order 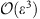, the LQTS reads


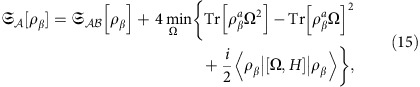


where we have defined 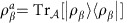. By differentiating the trace with respect to Ω, we determine the minimization condition for it, yielding


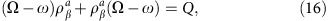


with 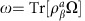 and 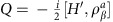, *H*′ being the analogous of *H* which acts on 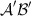 (by construction *H* |*ρ*_*β*_〉=*H*′|*ρ*_*β*_〉). Equation (16) explicitly implies that Ω does not depend on *ω*, which, without loss of generality, can be set to zero. Moreover, it enables to rewrite the LQTS in equation (15) as


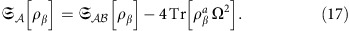


The solution of the operatorial equation (16) can be found by applying Lemma 1 presented at the end of this section, yielding


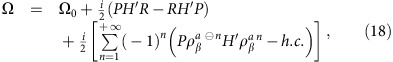


with Ω_0_ being an operator which anti-commutes with Ω, 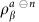 being the Moore–Penrose pseudoinverse of 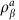 to the power *n*, *R* being the projector on kernel of 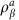, *P*=_*a*_−*R* being its complementary counterpart, and with *h.c.* denoting the hermitian conjugate term. By substituting this expression in equation (17), we finally get


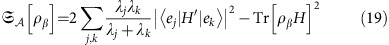


where 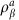
=∑_*i*_*λ*_*i*_|*e*_*i*_〉〈*e*_*i*_| is the spectral decomposition of 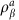, sharing the same spectrum with 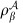. The expression above holds for both invertible and not invertible 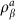. To the latter scenario belongs the case in which *H*=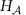
+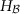, where one can easily prove that the LQTS reduces to the variance of the local Hamiltonian 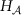, that is, 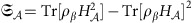 (notice that the non-zero eigenvalues of 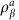 are 
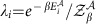
 which correspond to 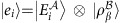, being 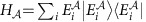, 
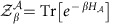
 and 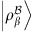 the purification of 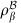 through the ancillary system 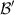). The expression above can also be rewritten as


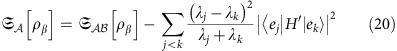


which can be cast into equation (5) by simply exploiting the fact that the system is symmetric with respect to the exchange of 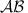 with 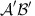.

It is finally useful to observe that LQTS can be also expressed in terms of the eigenvectors of 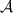, 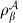
=∑_*i*_*λ*_*i*_|*g*_*i*_〉〈*g*_*i*_| as:


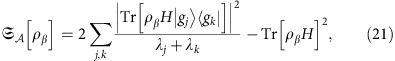


where we have used the Schmidt decomposition of |*ρ*_*β*_〉, with respect to bipartition 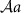,


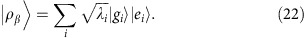


In particular, expression (21) can be exploited to numerically compute the LQTS, for instance when dealing with quantum many-body systems (see Fig. 3 and the discussion in the Supplementary Note 1).

*Lemma 1:* For any assigned operators *X*, *Y* satisfying the equation


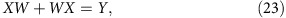


the following solution holds


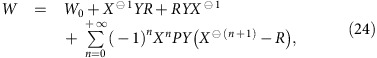


where 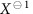 is the Moore Penrose pseudoinverse of *X*, *R* is the projector on kernel of *X*, *P*=−*R* ( indicates the identity matrix) and *W*_0_ is a generic operator which anti-commutes with *Y* (see also ref. 46). Furthermore if *X* and *Y* are Hermitian, equation (23) admits solutions which are Hermitian too: the latter can be expressed as


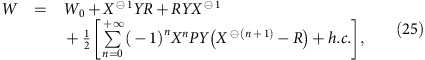


where now *W*_0_ is an arbitrary Hermitian operator which anti-commutes with *Y*.

Proof: Since (23) is a linear equation, a generic solution can be expressed as the sum of a particular solution plus a solution *W*_0_ of the associated homogeneous equation, that is, an operator which anti-commute with *X*,


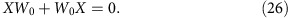


A particular solution *W* of equation (23) can be always decomposed as


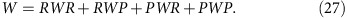


Notice that by definition, *RX*=*XR*=*O*, where *O* identifies the null operator. Multiplying (23) on both sides by *R*, one gets the condition *RYR*=*O*. The operator *W*, solution of equation (23), is defined up to its projection on the kernel subspace, that is


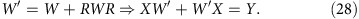


Therefore, without loss of generality we can set


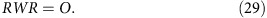


Multiplying equation (23) by 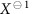 on the right side and repeating the same operation on the left side, we get:


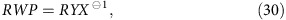



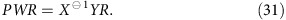


On the other hand, *PWP* satisfies the relation


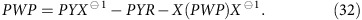


This equation can be solved recursively in *PWP* and gives


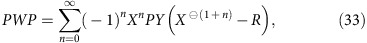


thus concluding the first part of the proof. The second part of the proof follows simply by observing that, if *X* and *Y* are Hermitian, and if *W* solves equation (23), then also its adjoint counterpart does. Therefore, for each solution *W* of the problem, we can construct an Hermitian one by simply taking (*W*+*W*^†^)/2.

### Second-order term corrections to LQTS

In the low-temperature regime (*β*→∞), we have computed the second-order correction term to the LQTS, that is of 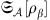 in equation (1), and found:


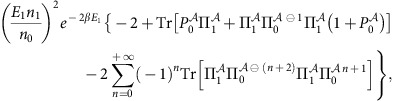


with *E*_*k*_≥*E*_1_ and where the series in *n* is meant to converge to 1/2 when 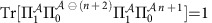, that is, 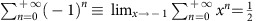. To vanish, this second-order correction term requires a stronger condition with respect to one necessary to nullify the first-order term in the LQTS, equation (7). It is given by 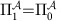, and corresponds to the requirement that the system ground state must be locally indistinguishable from the first excited level.

### Heat capacity in the two-level LZ scheme

**Figure 4:**
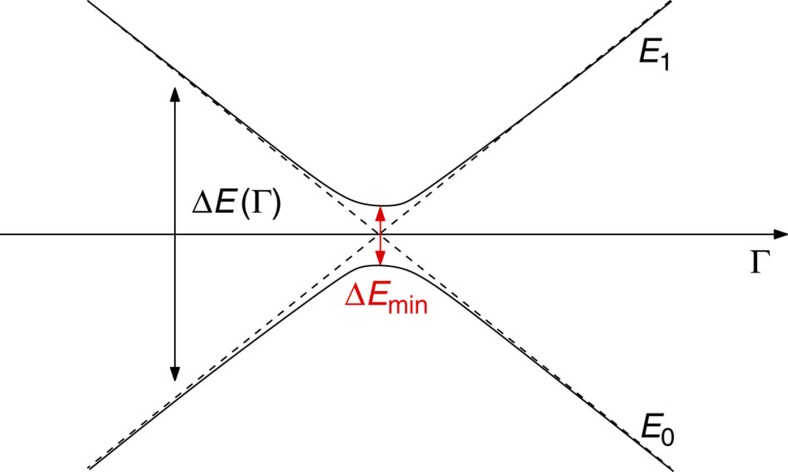
The two-level Landau–Zener model. A sketch of the behaviour of the two eigenenergies (*E*_1_, *E*_2_) as a function of some control parameter Γ. The gap Δ*E*=*E*_2_−*E*_1_ displays a pronounced minimum in correspondence of a given Γ_c_ value.

Here we discuss the simplified case in which only the ground state (with energy *E*_0_) and the first excited level (with energy *E*_1_) of the global system Hamiltonian *H* play a role. In particular, we are interested in addressing a situation where the ground-state energy gap Δ*E*≡*E*_1_−*E*_0_ may become very small, as a function of some external control parameter Γ (for example, the magnetic field or the system anisotropy). A sketch is depicted in Fig. 4, and refers to the so-called LZ model^42^. This resembles the usual scenario when a given many-body system is adiabatically driven, at zero temperature, across a quantum phase transition point.

In correspondence of some critical value Γ_c_, the gap is minimum. For a typical quantum many-body system, such minimum value Δ*E*_min_ tends to close at the thermodynamic limit and a quantum phase transition occurs (notice that Γ_c_ may depend on the system size). Hereafter, without loss of generality, we will assume *E*_0_=0 and take *E*_1_=Δ*E* so that the system heat capacity (3) reduces to:


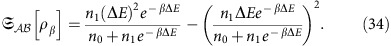


Here *n*_0_ and *n*_1_ are the degeneracy indexes associated to the levels *E*_0_ and *E*_1_, respectively. Notice that 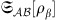 is always non-negative and exhibits a non-monotonic behaviour as a function of Δ*E*, at fixed *β*. Indeed it is immediate to see that 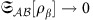 in both limits Δ*E*→0 and Δ*E*→+∞. For fixed *β*, *n*_0_ and *n*_1_, the heat capacity displays a maximum in correspondence of the solution of the transcendental equation


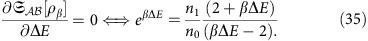


In particular, for *n*_0_=*n*_1_=1, the latter relation is fulfilled for 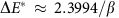, while for *n*_0_=2, *n*_1_=1, it is fulfilled for 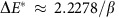.

**Figure 5:**
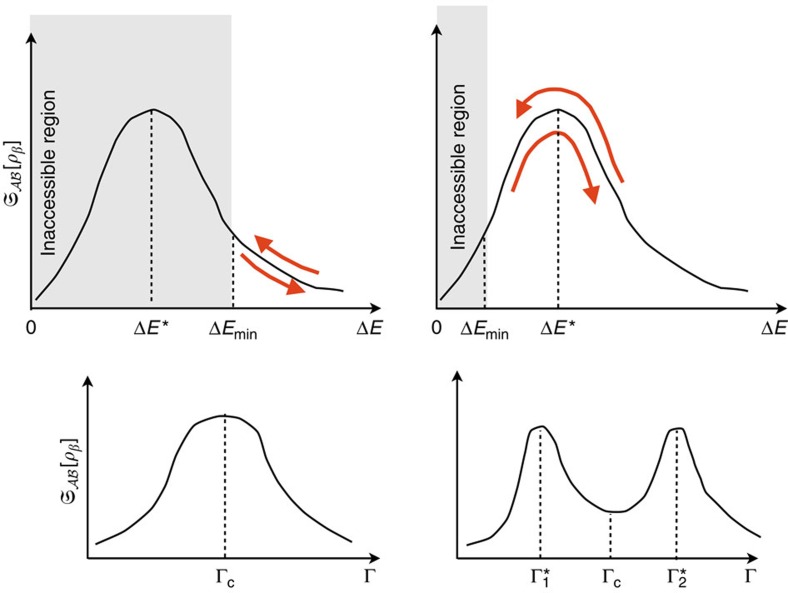
Heat capacity in the Landau–Zener model. The emergence of extremal points in the behaviour of 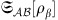 as a function of increasing control parameter Γ is associated to the relative size of Δ*E*_min_ with respect to ΔE*. The red arrows denote the changing of Δ*E* during a typical Landau–Zener protocol. One realizes that, if Δ*E*_min_>ΔE* one peak will appear (left), whereas if Δ*E*_min_<ΔE* two peaks will appear (right).

It turns out that the behaviour of the heat capacity as a function of increasing Γ in a two-level LZ scheme depends on the relative sizes of ΔE* and Δ*E*_min_, as pictorially shown in Fig. 5: (a) if Δ*E*_min_>ΔE*, then 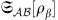 will exhibit a maximum in correspondence of Γ_c_; (b) if Δ*E*_min_<ΔE*, a maximum at 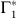 corresponding to Δ*E*=ΔE* will appear, followed by a local minimum at Γ_c_ and eventually by another maximum at 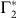 where the former condition occurs again. Since ΔE* is a function of β, and Δ*E*_min_ depends on the system size, the point of minimum gap can be signalled by a maximum or by a local minimum depending on the way the two limits *L*→+∞ (thermodynamic limit) and *β*→+∞ (zero-temperature limit) are performed. In the Supplementary Note 2, we explicitly address the two many-body Hamiltonians considered in the last subsection of the ‘Results' section, namely the Ising and the XXZ model (see the Supplementary Figs 3 and 4). Here in particular, we discussed the possible emergence of corrections to the low-temperature energy variance (34) when one takes into account the presence of the low-lying energy levels beyond the first excited one.

### Data availability

The data that support the findings of this study are available from the corresponding author upon request.

## Additional information

**How to cite this article:** De Pasquale, A. *et al.* Local quantum thermal susceptibility. *Nat. Commun.* 7:12782 doi: 10.1038/ncomms12782 (2016).

## Supplementary Material

Supplementary Information Supplementary Figures 1-4 and Supplementary Notes 1-2.

## Figures and Tables

**Figure 1:**
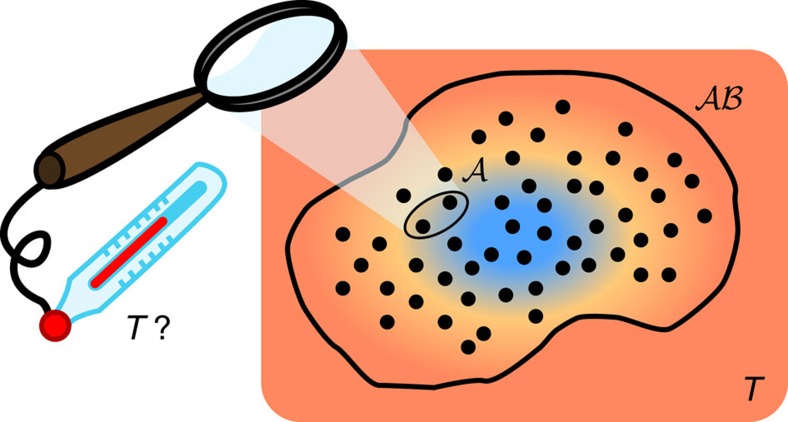
Temperature estimation via local measures. We propose the following operationally grounded strategy, which is embodied by the local quantum thermal susceptibility (LQTS) functional. A composite quantum system 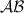 is in thermal equilibrium with a bath at temperature *T*. The LQTS measures the highest achievable accuracy in the estimation of *T* under the hypothesis to perform only local measurements on a given subsystem 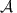 of 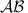.
