# Supplementary material for: Local quantum thermal susceptibility
Source: Nat Commun. 2016 Sep 29;7:12782. doi: 10.1038/ncomms12782 (PMC5056999; doi:10.1038/ncomms12782)
Supplement: Supplementary Information — Supplementary Figures 1-4 and Supplementary Notes 1-2. [file ncomms12782-s1.pdf]

SUPPLEMENTARY INFORMATION

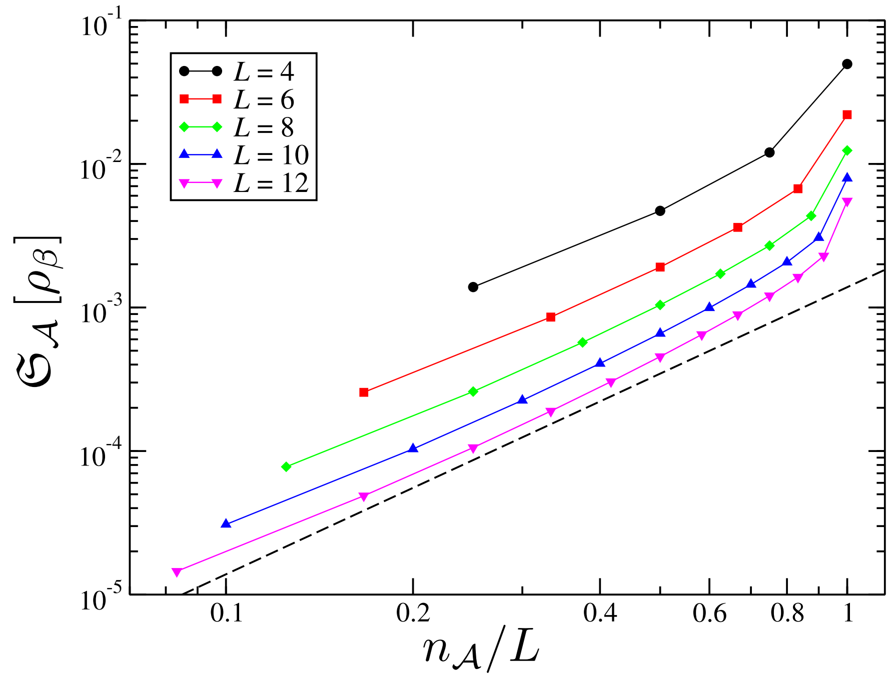

SUPPLEMENTARY FIGURE 1: **Analysis of the peak values of the LQTS for the Ising model.** Peak value of $\mathfrak{S}_{\mathcal{A}}[\rho_\beta]$ for the Ising model, as a function of the dimension of the measured subsystem $n_{\mathcal{A}}$. Different data sets are for various system lengths L. The dashed line denotes a power-law behaviour $\mathfrak{S}_{\mathcal{A}} \sim (n_{\mathcal{A}}/L)^2$, and is plotted as a guideline. The temperature is chosen as a function of the system size, as $\beta = 3L/4$.

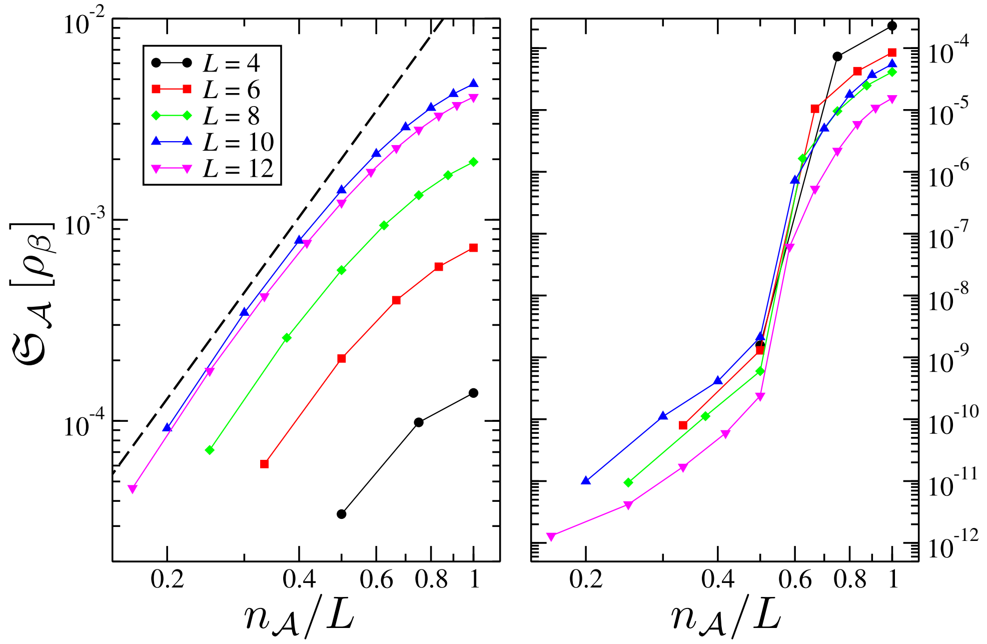

SUPPLEMENTARY FIGURE 2: **Analysis of the peak values of the LQTS for the XXZ model.** Same quantity and parameters as in the Supplementary Figure 1, but for the XXZ chain. The data in the two panels are for the minima of the LQTS close to the critical points $\Delta_{\mathrm{f}} = -1$ (left) and $\Delta_{\mathrm{af}} = +1$ (right). The dashed line in the left panel denotes the behaviour $\mathfrak{S}_{\mathcal{A}} \sim (n_{\mathcal{A}}/L)^3$ and is plotted as a guideline.

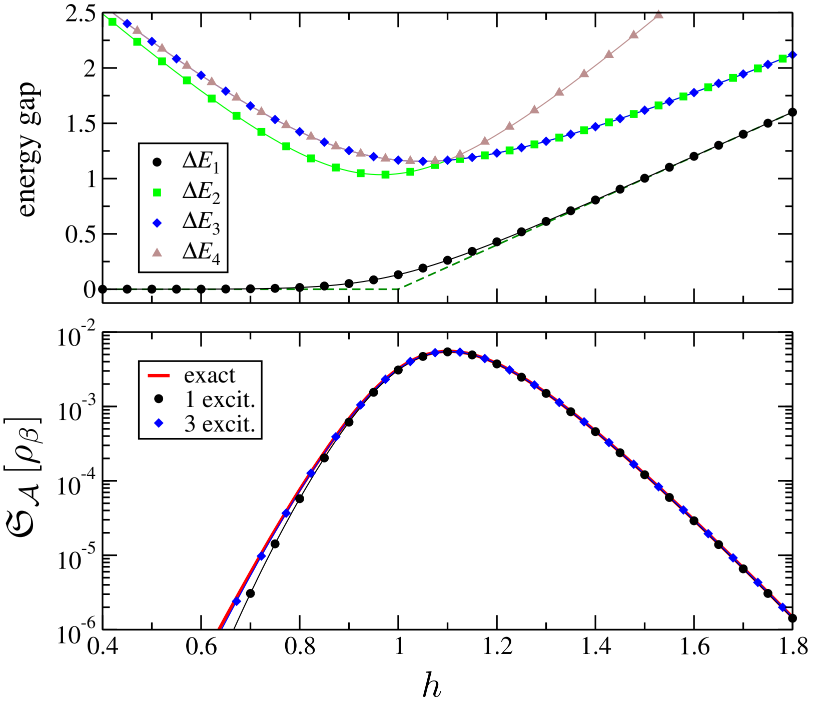

SUPPLEMENTARY FIGURE 3: **Heat capacity for the Ising chain, without breaking the $\mathbb{Z}_2$ symmetry**. The upper panel shows the first energy gaps $\Delta E_j = E_j - E_0$ as a function of the transverse field h. The dashed green line denotes the gap ΔE_1 in the thermodynamic limit. The lower panel shows the global quantum thermal susceptibility $\mathfrak{S}_{\mathcal{AB}}[\rho_\beta]$ for the full spectrum (red), and when taking into account the first eigenenergies only. Here we consider a chain with $L = 12$ and a temperature $\beta = 9$.

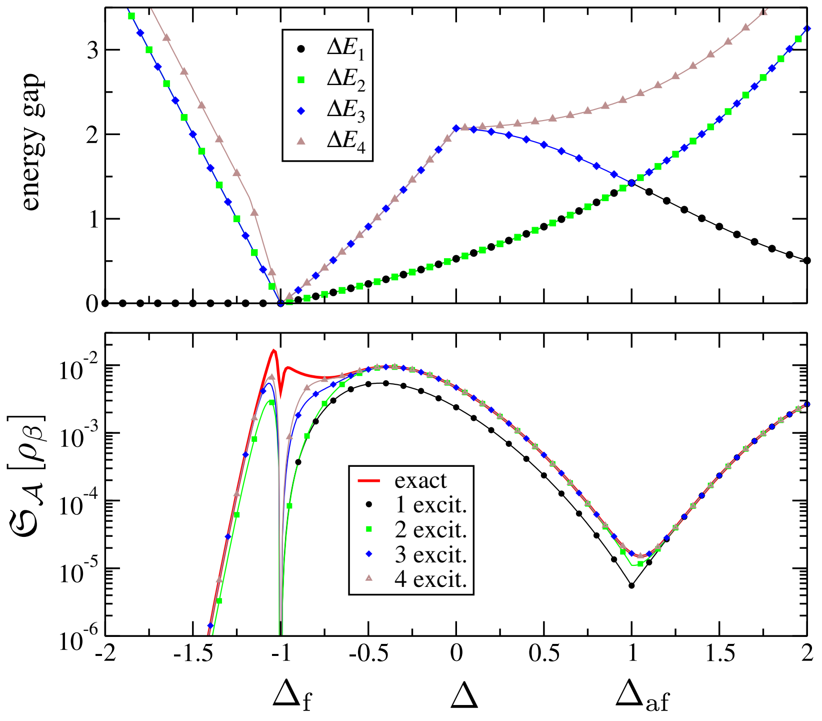

SUPPLEMENTARY FIGURE 4: **Heat capacity for the XXZ model.** Same quantities and parameters as in the Supplementary Figure 3, but for the XXZ chain in the full many-body Hilbert space of L spins.

Supplementary Note 1: Scaling of the LQTS with the dimension $n_{\mathcal{A}}$ of subsystem $\mathcal{A}$.

The local quantum thermal susceptibility (LQTS) for the subsystem $\mathcal{A}$ of a given system, defined as

$$\mathfrak{S}_{\mathcal{A}}[\rho_\beta] := 8 \lim_{\varepsilon\to 0} \frac{1-\mathcal{F}\big(\rho_\beta^{\mathcal{A}}, \rho_{\beta+\varepsilon}^{\mathcal{A}}\big)}{\varepsilon^2} \tag{1}$$

(see equation (1) in the main text), is a quantity which is tricky to be evaluated numerically. Apart from the exponential growth of the Hilbert space, extrapolating the limit $\varepsilon \to 0$ typically requires high accuracies in the diagonalization procedure. This would generally limit the study of local thermometry in the many-body context up to very small systems. The expression

$$\mathfrak{S}_{\mathcal{A}}[\rho_\beta] = 2\sum_{j,k} \frac{|\mathrm{Tr}[\rho_\beta H |g_j\rangle\langle g_k|]|^2}{\lambda_j+\lambda_k} - \mathrm{Tr}[\rho_\beta H]^2, \tag{2}$$

that we derived in equation (21) in the Methods of the main text, circumvents the latter problem and enables an easier manipulation of $\mathfrak{S}_{\mathcal{A}}[\rho_\beta]$. However one still needs the full spectrum of the reduced density matrix $\rho_\beta^{\mathcal{A}}$, since all its eigenvectors have to be contracted with the product of the global equilibrium state times the system Hamiltonian, $\rho_\beta H$. This makes the whole analysis not straightforward, even for free-fermion systems as is the case for the Ising model $H_{\rm Ising}$. For this reason we resort to an exact diagonalization technique.

Let us concentrate on the scaling of the LQTS with the size $n_{\mathcal{A}}$ of the measured subsystem for the Ising spin chain, defined by the Hamiltonian

$$H_{\rm Ising} = -J\sum_i \left[\sigma_i^x\sigma_{i+1}^x + h\sigma_i^z\right] \tag{3}$$

(equation (9) in the main text). The maximum of $\mathfrak{S}_{\mathcal{A}}[\rho_\beta]$ as a function of $n_{\mathcal{A}}$, and for different system sizes L is shown in the Supplementary Figure 1. As noticed in the main text within the LZ discussion, the peak is related to the appearance of the critical point, but deviations from its position at the thermodynamic limit ($h_{\rm c}=1$) are expected, due to the mutual interplay of finite-size and finite-temperature effects. To balance them, we choose a size-dependent temperature $\beta = 3L/4$. While with exact diagonalization we cannot go beyond the study of systems with size $L=12$, our data suggest a power-law behaviour of the peak value at least for very small subsystems, scaling as

$$\mathfrak{S}_{\mathcal{A}}[\rho_\beta] \sim (n_{\mathcal{A}}/L)^\alpha, \tag{4}$$

with an exponent $\alpha \approx 2$.

We performed the same analysis also for the XXZ chain, defined by the Hamiltonian

$$H_{\rm XXZ} = J\sum_i \left[(\sigma_i^x\sigma_{i+1}^x + \sigma_i^y\sigma_{i+1}^y) + \Delta\,\sigma_i^z\sigma_{i+1}^z\right] \tag{5}$$

(equation (10) in the main text), and focused on the behaviour of $\mathfrak{S}_{\mathcal{A}}[\rho_\beta]$ around the two critical points of the model, in correspondence to the two local minima. As we did above, we fix a size-dependent temperature $\beta = 3L/4$ and study systems of size up to $L=12$. In the left panel of Supplementary Figure 2, we concentrate on the ferromagnetic point at $\Delta_{\rm f}=-1$. Similarly to the critical point of the Ising model, a power-law scaling of the type in equation (4) seems to emerge at small $n_{\mathcal{A}}$, with an exponent $\alpha \approx 3$. The scaling analysis at the antiferromagnetic point around $\Delta_{\rm af}=1$ is less clear and probably requires larger system sizes (right panel). We notice the appearance of a cusp-like feature at $n_{\mathcal{A}} = L/2$, which reflects the different behaviour of $\mathfrak{S}_{\mathcal{A}}[\rho_\beta]$ for $\Delta \approx \Delta_{\rm af}$, depending on whether $n_{\mathcal{A}} \le L/2$ or $n_{\mathcal{A}} > L/2$, as is clearly visible in the right panel of Figure 3 in the main text. We have to stress that the departure of the two bunches of curves becomes more evident at large values of β, while it tends to disappear when increasing the temperature.

Supplementary Note 2: Analysis of the heat capacity by considering only the first excited levels.

The heat capacity of the global system is quantified by the variance of the energy,

$$\mathfrak{S}_{\mathcal{AB}}[\rho_\beta] = \mathrm{Tr}[\rho_\beta H^2] - \mathrm{Tr}[\rho_\beta H]^2\,, \tag{6}$$

and it can be calculated easier than the LQTS, since it does not involve any partial tracing over a portion of the system and depends only on the spectral properties. In particular, at low temperatures $\beta \to +\infty$, the relevant contributions to $\mathfrak{S}_{\mathcal{AB}}[\rho_\beta]$ will be provided only by the first low-lying energy levels, as discussed within the Landau-Zener (LZ) context. Here we provide an analysis of the role of such excited states in the two many-body systems that we address in this work.

We start from the Ising chain (3). The low-lying spectrum is shown in the upper panel of Supplementary Figure 3, where we plot the gaps $\Delta E_j = E_j - E_0$ between the ground-state energy E_0 and those of the first excited states E_j, $j > 0$. Since we are considering the full Hilbert space of the system, for $L \to \infty$ the ground state in the ferromagnetic side ($|h| < h_c$) is doubly degenerate, while in the paramagnetic side ($|h| > h_c$) a gap opens up monotonically as $\Delta E_1 = 2|h - h_c|$. At finite values of L (data with symbols), the double degeneracy survives only at $h = 0$ and Δ_1 monotonically increases with $h > 0$.

The scenario emerging by only keeping contributions to $\mathfrak{S}_{\mathcal{AB}}[\rho_\beta]$ coming from the ground state and the first excited state, while neglecting any other excited level, is quite clear. In the thermodynamic limit and for finite β, the heat capacity is rigorously zero for $h < h_c$ and is finite for $h > h_c$, exhibiting a non-monotonic behaviour. The position h_c^* of the maximum depends on the value of β and shifts toward $h_c = 1$ as long as $\beta \to \infty$. Note however that $\mathfrak{S}_{\mathcal{AB}}[\rho_\beta] \overset{\beta\to\infty}{\longrightarrow} 0$. In summary, the position of the maximum depends on a competition between the following two effects: *i*) decreasing L tends to shift the peak toward the ferromagnetic phase ($h_c^* < h_c$); *ii*) decreasing β tends to shift the peak toward the paramagnetic phase ($h_c^* > h_c$).

In the bottom panel of Supplementary Figure 3 we observe that, keeping only the ground and the first excited level in the computation of the energy variance (6), already gives an excellent approximation to the exact value of $\mathfrak{S}_{\mathcal{AB}}[\rho_\beta]$. Therefore the above situation effectively applies at large β values. In the presence of a symmetry-breaking mechanism, one would find a situation analogous to the LZ scheme: the presence of a maximum in proximity of h_c would not be guaranteed, and a local minimum may appear (according to the values of L and β).

Let us now consider the XXZ-Heisenberg chain (5). Analogously as above, the upper panel of Supplementary Figure 4 displays the first energy levels in the full Hilbert space (we do not break the symmetry associated to the conservation of the global magnetization along the z axis). For $\Delta < \Delta_f$, the ground state is fully polarized along z, and presents a double degeneracy at any length. At $\Delta = \Delta_{af}$ we see a cusp in the ground-state energy gap that is due to a level crossing (this feature persists at larger sizes L). This scenario characterizes the contribution to the heat capacity coming only by the ground state and the first excited state (black line in the bottom panel). In particular we see that, in this approximation, $\mathfrak{S}_{\mathcal{AB}}[\rho_\beta]$ is zero for $\Delta < \Delta_f$, while it becomes finite at $\Delta > \Delta_f$. On the other side, the Δ_{af} is signaled by a cusp, which displays a minimum, and which raises as a consequence of the cusp in the ground-state energy gap.

Contrary to the Ising model, if also other excited levels are taken into account, non-negligible corrections to the energy variance appear (see the bottom panel of Supplementary Figure 4). In particular we note the emergence of a minimum around Δ_f, due to the fact that $\mathfrak{S}_{\mathcal{AB}}[\rho_\beta]$ becomes finite also for $J_z < \Delta_f$. The corner point at Δ_{af} is smeared into a local minimum with a continuous derivative. The positions of the two minima are influenced by finite-size and finite-temperature effects.
